# *Cannabis sativa* L. (var. *indica*) Exhibits Hepatoprotective Effects by Modulating Hepatic Lipid Profile and Mitigating Gluconeogenesis and Cholinergic Dysfunction in Oxidative Hepatic Injury

**DOI:** 10.3389/fphar.2021.705402

**Published:** 2021-12-21

**Authors:** Ochuko L. Erukainure, Motlalepula G. Matsabisa, Veronica F. Salau, Sunday O. Oyedemi, Omolola R. Oyenihi, Collins U. Ibeji, Md. Shahidul Islam

**Affiliations:** ^1^ Department of Pharmacology, School of Clinical Medicine, Faculty of Health Sciences, University of the Free State, Bloemfontein, South Africa; ^2^ Department of Biochemistry, School of Life Sciences, University of KwaZulu-Natal, (Westville Campus), Durban, South Africa; ^3^ Department of Pharmacology, School of Science and Technology, Nottingham Trent University, Nottingham, UK; ^4^ Department of Pure and Industrial Chemistry, Faculty of Physical Sciences, University of Nigeria, Nsukka, Nigeria

**Keywords:** cannabidiol, gluconeogenesis, hepatic injury, oxidative stress, *Cannabis sativa* L.

## Abstract

*Cannabis sativa* L. is a crop utilized globally for recreational, therapeutic, and religious purposes. Although considered as an illicit drug in most countries, *C. sativa* until recently started gaining attention for its medicinal application. This study sought to investigate the hepatoprotective effect of *C. sativa* on iron-mediated oxidative hepatic injury. Hepatic injury was induced *ex vivo* by incubating hepatic tissues with Fe^2+^, which led to depleted levels of reduced glutathione, superoxide dismutase, catalase and ENTPDase activities, triglyceride, and high-density lipoprotein–cholesterol (HDL-C). Induction of hepatic injury also caused significant elevation of malondialdehyde, nitric oxide, cholesterol, and low-density lipoprotein–cholesterol (LDL-C) levels while concomitantly elevating the activities of ATPase, glycogen phosphorylase, glucose-6-phosphatase, fructose-1,6-bisphosphatase, amylase, and lipase. Treatment with the hexane, dichloromethane (DCM), and ethanol extracts of *C. sativa* leaves significantly (*p* < 0.05) reversed these levels and activities to almost near normal. However, there was no significant effect on the HDL-C level. The extracts also improved the utilization of glucose in Chang liver cells. High-performance liquid chromatography (HPLC) analysis showed the presence of phenolics in all extracts, with the ethanol extract having the highest constituents. Cannabidiol (CBD) was identified in all the extracts, while Δ-9-tetrahydrocannabinol (Δ-9-THC) was identified in the hexane and DCM extracts only. Molecular docking studies revealed strong interactions between CBD and Δ-9-THC with the *β*2 adrenergic receptor of the adrenergic system. The results demonstrate the potential of *C. sativa* to protect against oxidative-mediated hepatic injury by stalling oxidative stress, gluconeogenesis, and hepatic lipid accumulation while modulating cholinergic and purinergic activities. These activities may be associated with the synergistic effect of the compounds identified and possible interactions with the adrenergic system.

## Introduction

The roles of the liver in the metabolic functions of the body are well documented (Abdel-Salam et al., 2014; Seif, 2016). The liver is among the prominent organs in the body required for maintaining and regulating the body’s homeostasis, thus playing a key role in the detoxification and excretion of endogenous and exogenous compounds as well as carbohydrate, protein, and fat metabolism (Pandit et al., 2012; Seif, 2016). The liver has also been reported to play a principal role in iron homeostasis as well as storing excess iron (Anderson and Frazer, 2005; Rishi and Subramaniam, 2017).

Iron is regarded as one of the essential elements owing to its roles in the body leading to optimal health. It is involved in several metabolic reactions and is a cofactor for enzymes involved in most physiological functions due to its ability to assume two different ionic states [ferrous (Fe^2+^) and ferric (Fe^3+^)] (Rishi and Subramaniam, 2017). This ability also makes it an influential regulator of the cell’s redox homeostatic state (Andrews, 1999; Erukainure et al., 2017). However, dysregulation of iron homeostasis leading to excessive increase in hepatic (liver) iron storage has been implicated in exacerbated production of free radicals (Anderson and Shah, 2013). This increased production causes an imbalance in the redox state owing to suppression of the tissues’ intrinsic antioxidant defense system, which consequently leads to oxidative hepatic injury. Oxidative injury has been implicated as an important mechanism in the pathogenesis and progression of several hepatic diseases such as cirrhosis, hepatitis, hepatocellular carcinoma, and fibrosis (Anderson and Shah, 2013; Milic et al., 2016).

Medicinal plants have been utilized in ancient history in the folkloric treatment and management of liver diseases and several ailments such as diabetes, cancer, and malaria. Folkloric medicine and its method of treatment are often correlated with the tradition and culture of the community where the plants are indigenous, thus contributing to the cultural heritage and health system of the community. The efficacies of these plants can be associated with their phytochemical and nutritional components (Chukwuma et al., 2019). Among such plants is *Cannabis sativa* L.

A member of the *Cannabis* genus and the Cannabaceae family, *C. sativa* popularly referred to as marijuana, Indian hemp, and weed is an annual herbaceous plant utilized for recreational, therapeutic, and religious purposes as well as for food (Kuddus et al., 2013; Bonini et al., 2018). It is globally widespread, with Africa reported for its highest production, as it accounts for over 25% of the global production (UNODC, 2012). Its major phytochemical constituents consist of phytocannabinoids, which are a class of C21–C22 terpenophenolic compounds, with tetrahydrocannabinol (THC), cannabinol (CBN), and cannabidiol (CBD) being the most common (Andre et al., 2016). Its use in traditional medicine has been reported for treating a number of ailments including diabetes, liver diseases, cancer, and neurological disorders particularly in Chinese and Indian traditional medicine (Russo, 2005; Brand and Zhao, 2017; Ryz et al., 2017). These are supported by a number of studies that have shown the therapeutic properties of *C. sativa* and its constituents, which include anticancer activity (Guzman, 2003), antidiabetic activity (Ren et al., 2016), pain suppression (Whiting et al., 2015), anti-neurodegeneration activity (Aso and Ferrer, 2016), sleep management (Ramar et al., 2018), protection from liver diseases (Musa et al., 2012; Hussein et al., 2014), anti-colitis activity, and anti-epilepsy activity (Fusar-Poli et al., 2009). Despite its medicinal properties, *C. sativa* is considered an illicit drug in most countries but has recently started gaining attention for its medicinal application.

This study sought out to investigate the hepatoprotective potential of *C. sativa* leaves by determining their ability to suppress oxidative stress and hepatic lipid level, as well as their effect on carbohydrate metabolism, purinergic activities, and cholinergic dysfunction in ferric-mediated oxidative hepatic injury.

## Materials and Methods

### Plant Material

This research has been undertaken with the approval (Permit No. POS 248/2019/2020) of the South African Health Products Regulatory Authority to conduct, collect, possess, transport, and store cannabis plant, plant parts, and products for research purposes. The study was also conducted to collect cannabis plants in Lesotho under the permit (Permit #: 01/LS/2019/10/02-01).

The leaves of *C. sativa* were obtained from Mohale’s Hoek District, Lesotho (GPS coordinates: 30.333,776″S and 27.651,201″E). The plant was authenticated by the Geo Potts Herbarium at the University of the Free State, Bloemfontein 9300, South Africa, and assigned the voucher number BLFU MGM 0018. This was further verified in the plant list online database (http://www.theplantlist.org/tpl1.1/search?q=Cannabis+Sativa+L).

The leaves were air-dried and pulverized to dry powder before undergoing sequential extraction using solvents of increasing polarity, namely, hexane, dichloromethane (DCM), and ethanol, for 48 h with gentle agitation of 100 rpm at room temperature. Each solvent was decanted and concentrated *in vacuo* using an R-215 rotary evaporator (Buchi, Flawil, Switzerland). The extracts were collected in glass vials and stored in the dark at ambient room temperature.

### Animals

Five male albino rats (Sprague Dawley strain) that weighed between 180 and 200 g were obtained from the Biomedical Research Unit (BRU), University of KwaZulu-Natal, Durban, South Africa. The animals were housed in plastic cages, fed on pelletized chows, and provided with water *ad libitum*, all under a 12-h light-dark cycle of natural photoperiod.

Rats were deprived of food (overnight fasting) 12 h prior to sacrifice. They were humanely sacrificed by euthanizing with isoform. Their livers were harvested, and bloodstains were removed by rinsing in 0.9% NaCl solution and then homogenized in 50 mM of sodium phosphate buffer, pH 7.5 (with 1% Triton X-100). The homogenized samples were then centrifuged at 15,000 rpm for 10 min at 4°C. The supernatant was collected and used immediately for *ex vivo* studies.

Rats were maintained under the approved guidelines of the Animal Ethics Committee of the University of KwaZulu-Natal, Durban, South Africa, and the study received ethical approval from the University of KwaZulu-Natal Animal Ethics Committee (Protocol approval number: AREC/22/019D).

### Induction of Oxidative Injury

Oxidative injury was induced *in vitro* using earlier established protocols with slight modifications (Akomolafe et al., 2013; Kizil et al., 2010). Briefly, 100 µl of the hepatic tissue supernatant was incubated with 15–240 μg/ml each of hexane, DCM, and ethanol extracts in the presence of 0.1 mM of FeSO_4_ at 37°C for 30 min. CBD was used as a reference drug, as it is the main non-psychoactive compound in *C. sativa*. Reactions without the extract or reference drug represented negative control (untreated). The normal control consisted of hepatic tissue homogenates without extracts, reference drugs, and FeSO_4_. The choice of doses was based on previous reports on the hepatoprotective properties of medicinal plants (Sanni et al., 2018).

### Antioxidative Activities

The hepatic tissue was analyzed for levels of reduced glutathione (GSH) (Ellman, 1959), catalase and superoxide dismutase (SOD) activities (Chance and Maehly, 1955; Kakkar et al., 1984), and malondialdehyde (MDA) level (Chowdhury and Soulsby, 2002).

### Nitric Oxide Level

The level of nitric oxide (NO) in the hepatic tissues was determined using the Griess method (Tsikas, 2005; Erukainure et al., 2019).

### Cholinergic Enzyme Activities

The cholinergic status of the hepatic tissues was estimated using Ellman’s procedure to analyze the activity of acetylcholinesterase (Ellman et al., 1961).

### Purinergic Enzyme Activities

The purinergic activities of the hepatic tissues were analyzed by determining the adenylpyrophosphatase (ATPase) (Adewoye et al., 2000; Erukainure et al., 2017) and ecto-nucleoside triphosphate diphosphohydrolase (ENTPDase) (Akomolafe et al., 2017) activities.

### Gluconeogenic Enzyme Activities

The hepatic tissues were examined for gluconeogenic activities by determining the activities of glycogen phosphorylase (Cornblath et al., 1963; Balogun and Ashafa, 2017), glucose-6-phosphatase (Mahato et al., 2011; Erukainure et al., 2017), and fructose-1,6-bisphosphatase (Gancedo and Gancedo, 1971; Balogun and Ashafa, 2017) in the supernatants.

### Hepatic Amylase Activity

The α-amylase activity of the hepatic tissues was determined by employing an earlier established method (Oboh et al., 2017).

### Hepatic Lipase Activity

The lipase activity of the hepatic tissues was determined by employing a previously modified protocol (Kim et al., 2010; Erukainure et al., 2020).

### Hepatic Lipid Profile

The lipid profile of the hepatic tissues was analyzed using a modified method described earlier (Erukainure et al., 2020). Briefly, hepatic tissues were incubated alongside 0.1 mM of FeSO_4_ and 240 μg/ml of the plant extracts or reference drug at 37°C overnight. The samples were thereafter centrifuged at 15,000 rpm for 10 min at 4°C. The tissue supernatants were decanted and used immediately to determine the total cholesterol, triglycerides (TGs), and high-density lipoprotein–cholesterol (HDL-C) of the hepatic tissues *via* an Automated Chemistry Analyzer (Labmax Plenno, Labtest Co. Ltd., Lagoa Santa, Brazil) with commercial assay kits according to the manufacturer’s manual.

### Glucose Utilization in Chang Liver Cells

Chang liver cells (ATCC^®^ CCL-13™) were obtained from the American Type Culture Collection (ATCC^®^), Manassas, VA, United States. The cells were seeded into flat-bottom 96-well culture plates (NUNC, Roskilde, Denmark) at a density of 6,000 cells/well in a volume of 200 µl/well of growth medium. After 72 h, 10 µl of the plant extracts (250 μg/ml) or metformin (20 µM) in Roswell Park Memorial Institute (RPMI) 1640 media (Helm AG, Hamburg, Germany) was added to the 200 µl medium already in the well to give a final concentration of 12.5 μg/ml and 1 µM for the plant extracts and metformin, respectively. They were thereafter subjected to induction for 48 h. After incubation, the growth medium was aspirated from the wells and replaced with 100 µl of freshly prepared incubation buffer (RPMI 1640 diluted with phosphate-buffered saline (PBS) to 8 mM of glucose with 0.1% (w/v) of bovine serum albumin (BSA; Roche Diagnostics, Mannheim, Germany)) with and/or without metformin or the extracts. The cells were further incubated for 3 h at 37°C. A volume of 50 μl of the reaction mixture was mixed with 200 μl of glucose oxidase reagent (SERA-PAK Plus, Bayer, Leverkusen, Germany) in a 96-well plate and incubated for 15 min at 37°C. Absorbance was read at 510 nm using a microplate reader. Glucose utilization was calculated by subtracting the amount of glucose left in the medium from the amount given at time 0 (Oyedemi et al., 2013).

### High-Performance Liquid Chromatography of Plant Extracts

High-performance liquid chromatography (HPLC)–diode array detection analysis of the extracts (hexane, DCM, and ethanol) was carried out with an Agilent 1100 series (Agilent, Waldbronn, Germany) instrument equipped with a photodiode array, an autosampler, a column thermostat, and a degasser. The stationary phase consisted of A Phenomenex: Luna 5 µm C_18_ 2) (150 × 4.6 mm; 5 μm particle size) column. Water containing 0.1% of formic acid (A) and acetonitrile (B) served as mobile phases at a flow rate of 1 ml/min. The gradient elution was as follows: initial ratio 95% A:5% B, maintained for 10 min, changed to 90% A:10% B in 10 min, changed to 70% A:30% B in 10 min, to 50% A:50% B in 10 min, maintained for 0.5 min, and back to initial ratio in 0.5 min. The temperature was set to 30°C, injection volume was 20.0 μl, and chromatograms were recorded at 254 nm (Erukainure et al., 2020).

### Molecular Docking Studies

Molecular docking was performed to determine the binding energy of THC and CBD with cannabinoid receptors. The 3D structure was retrieved from the cholesterol-bound form of human *β*2 adrenergic receptor (PDB access code: 3D4S (Hanson et al., 2008)) with a resolution of 2.8 Å. The suitable grid box was determined using AutoDock tools (Sanner, 1999). The structures of the ligands were retrieved from PUBMED and optimized using Gaussian 09 (Frisch et al., 2009); this was done to obtain minimized conformation. The determined dimension was X = 30 Y = 30 Z = 30 with 1.00 Å as the grid spacing. The optimum binding site for the ligand (Yang et al., 2013) was determined using the Lamarckian genetic algorithm method. Gasteiger charges were computed using the AutoDock Tools graphical user interface supplied by MGL Tools (Morris et al., 2009).

### Statistical Analysis

Data were analyzed by one-way ANOVA and presented as mean ± SD. Statistical significance was established at *p* < 0.05 using Tukey’s honestly significant difference (HSD)–multiple range post-hoc test. Statistical analyses were done using IBM Statistical Package for the Social Sciences (SPSS) for Windows, version 23.0 (IBM Corp., Armonk, NY, USA).

## Results and Discussion

**FIGURE 1:**
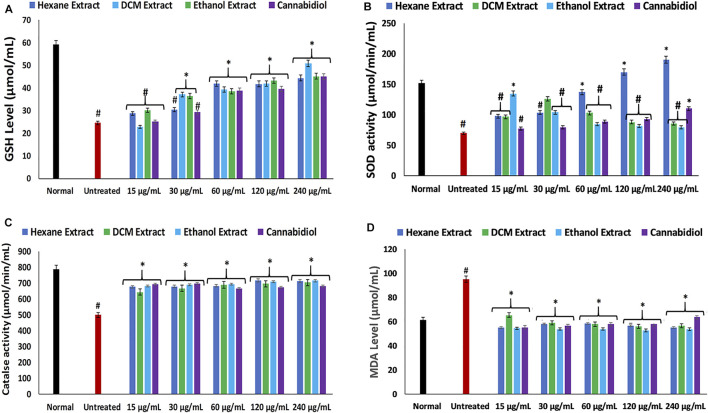
Effect of *Cannabis sativa* on **(A)** GSH level, **(B)** SOD, **(C)** catalase, and **(D)** MDA level in oxidative hepatic injury. Data = mean ± SD; *n* = 3. *Statistically significant (*p* < 0.05) compared with untreated tissues; #statistically significant (*p* < 0.05) compared with normal tissues. Normal: liver tissues not treated with FeSO_4_ and/or *C. sativa*. Untreated: liver tissues treated with FeSO_4_ only. GSH, reduced glutathione; SOD, superoxide dismutase; MDA, malondialdehyde.

**FIGURE 2:**
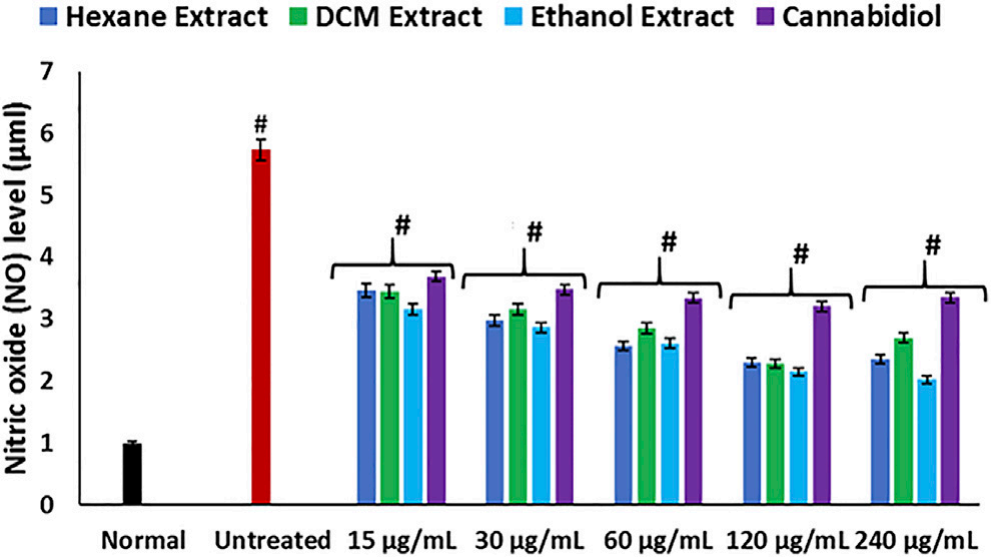
Effect of *Cannabis sativa* on NO level in oxidative hepatic injury. Data = mean ± SD; *n* = 3. *Statistically significant (*p* < 0.05) compared with untreated tissues; #statistically significant (*p* < 0.05) compared with the normal tissues. Normal: liver tissues not treated with FeSO_4_ and/or *C. sativa*. Untreated: liver tissues treated with FeSO_4_ only.

In the present study, the induction of oxidative injury in isolated rat hepatic tissues was established by incubating with FeSO_4_ in the presence of *C. sativa* extracts (hexane, DCM, and ethanol) and CBD. Induction of oxidative injury significantly depleted GSH level, SOD, and catalase activities, while concomitantly elevating MDA and NO levels as shown in Figure 1A–D; Figure 2, thus signifying an occurrence of oxidative stress and proinflammation on incubation of hepatic tissues with FeSO_4_. Oxidative stress has been reported in the pathogenesis and progression of hepatotoxicity and liver diseases (Zhu et al., 2012; Cichoż-Lach and Michalak, 2014), with a clear-cut in studies that reported increased oxidative stress markers in alcoholic hepatitis (Lu et al., 2008; Zhu et al., 2012). Treatment with *C. sativa* significantly reversed these activities and levels and was comparable with the active compound CBD, which served as a standard drug. These effect reversals indicate an ameliorative effect of *C. sativa* on hepatic oxidative stress. This is in tune with earlier reports on the protective properties of *C. sativa* on liver diseases (Musa et al., 2012; Hussein et al., 2014).

**FIGURE 3:**
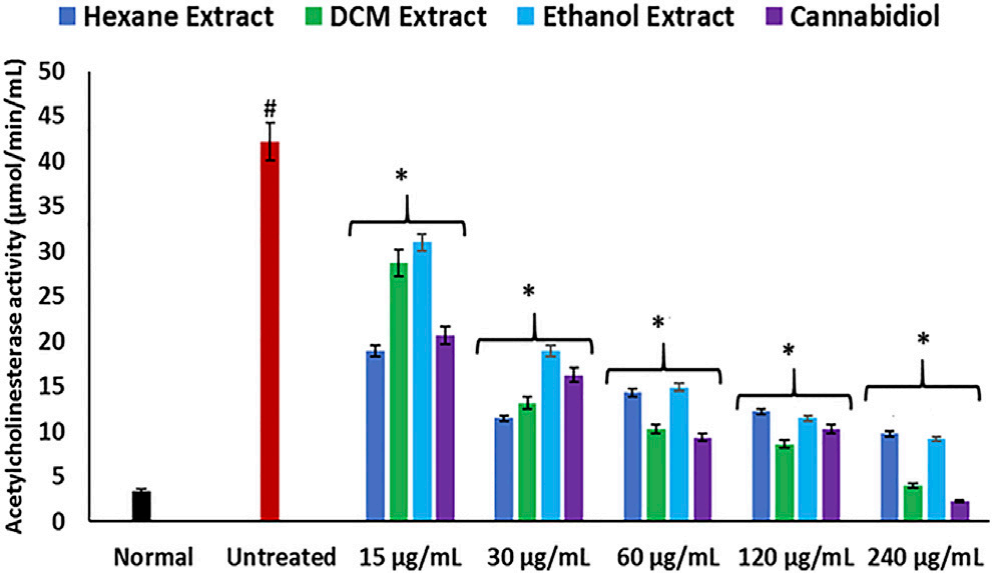
Effect of *Cannabis sativa* on acetylcholinesterase activity in oxidative hepatic injury. Data = mean ± SD; *n* = 3. *Statistically significant (*p* < 0.05) compared with untreated tissues; #statistically significant (*p* < 0.05) compared with normal tissues. Normal: liver tissues not treated with FeSO_4_ and/or *C. sativa*. Untreated: liver tissues treated with FeSO_4_ only.

Hepatic cholinergic dysfunction characterized by increased acetylcholinesterase activity has been associated with the pathogenesis of hepatotoxicity, as it triggers inflammation of the hepatocytes (García-Ayllón et al., 2012; García-Ayllón et al., 2006). The increased acetylcholinesterase activity occurrence on induction of oxidative injury (Figure 3) corroborates the occurrence of oxidative stress (Figures 1A–D) and elevated NO level (Figure 2), as a rise in oxidative stress and proinflammation has been implicated in increased acetylcholinesterase activity (Melo et al., 2003; Shaked et al., 2009). The reduced activity on treatment with *C. sativa* therefore indicates a protective effect of the extracts against cholinergic dysfunction in oxidative hepatotoxicity.

**FIGURE 4:**
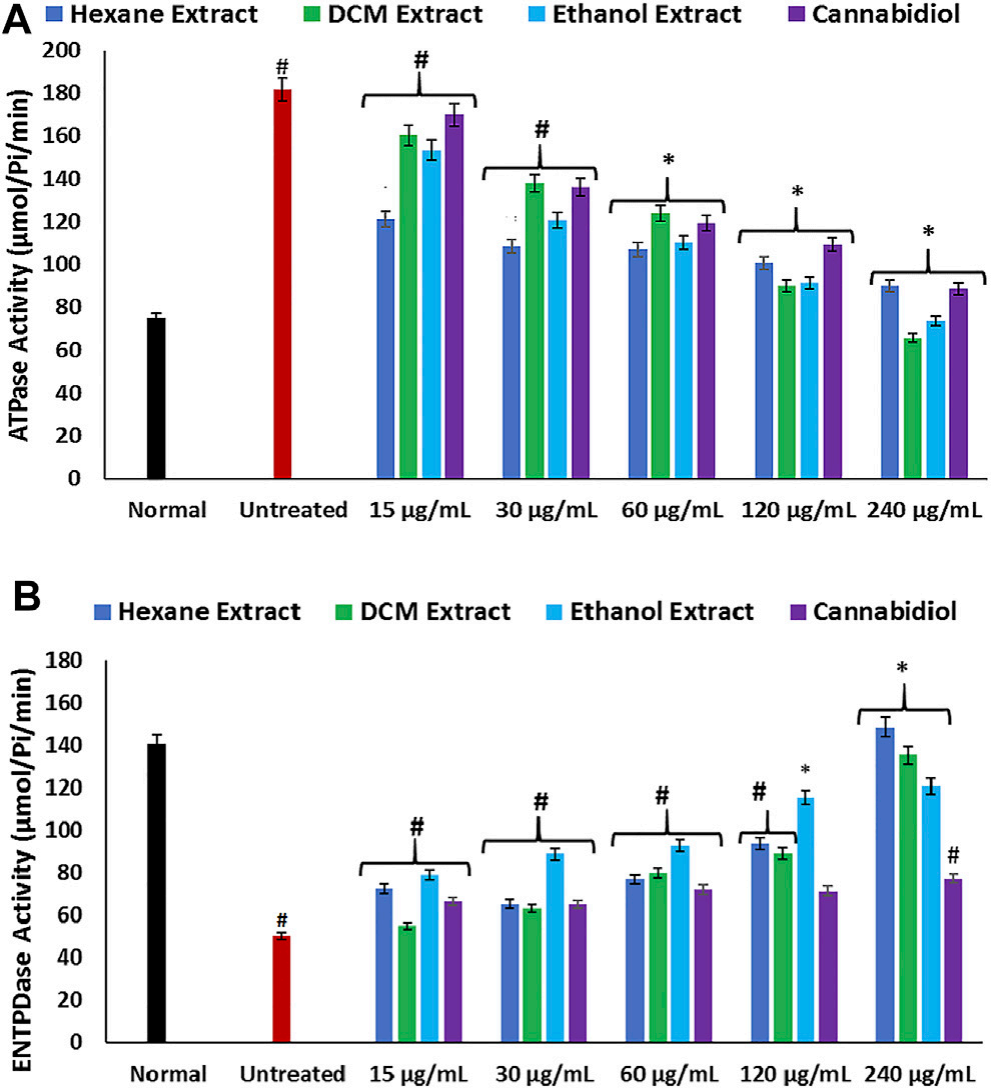
Effect of *Cannabis sativa* on **(A)** ATPase and **(B)** ENTPDase activities in oxidative hepatic injury. Data = mean ± SD; *n* = 3. *Statistically significant (*p* < 0.05) compared with untreated tissues; #statistically significant (*p* < 0.05) compared with normal tissues. Normal: liver tissues not treated with FeSO_4_ and/or *C. sativa*. Untreated: liver tissues treated with FeSO_4_ only. ATPase, adenylpyrophosphatase; ENTPDase, ecto-nucleoside triphosphate diphosphohydrolase.

Disturbances in hepatic purinergic enzyme activities have been implicated in hepatotoxicity (Sun et al., 2013). These enzymes catalyze phospho-hydrolysis of adenosine triphosphate (ATP) and adenosine monophosphate (AMP) to release the endogenous signaling nucleotide (Akinyemi et al., 2017; Bagatini et al., 2018). In this study, induction of oxidative injury led to a significant elevation of ATPase activity, with concomitant depletion of ENTPDase activity as depicted in Figures 4A,B. The increased ATPase activity portrays a depleted hepatic ATP activity, while the depleted ENTPDase activity depicts depleted levels of adenosine (Salau et al., 2019). There was a significant reversal of these activities on treatment with *C. sativa* extracts, thus indicating a modulatory effect of the extracts on purinergic activities in oxidative-mediated hepatotoxicity.

**FIGURE 5:**
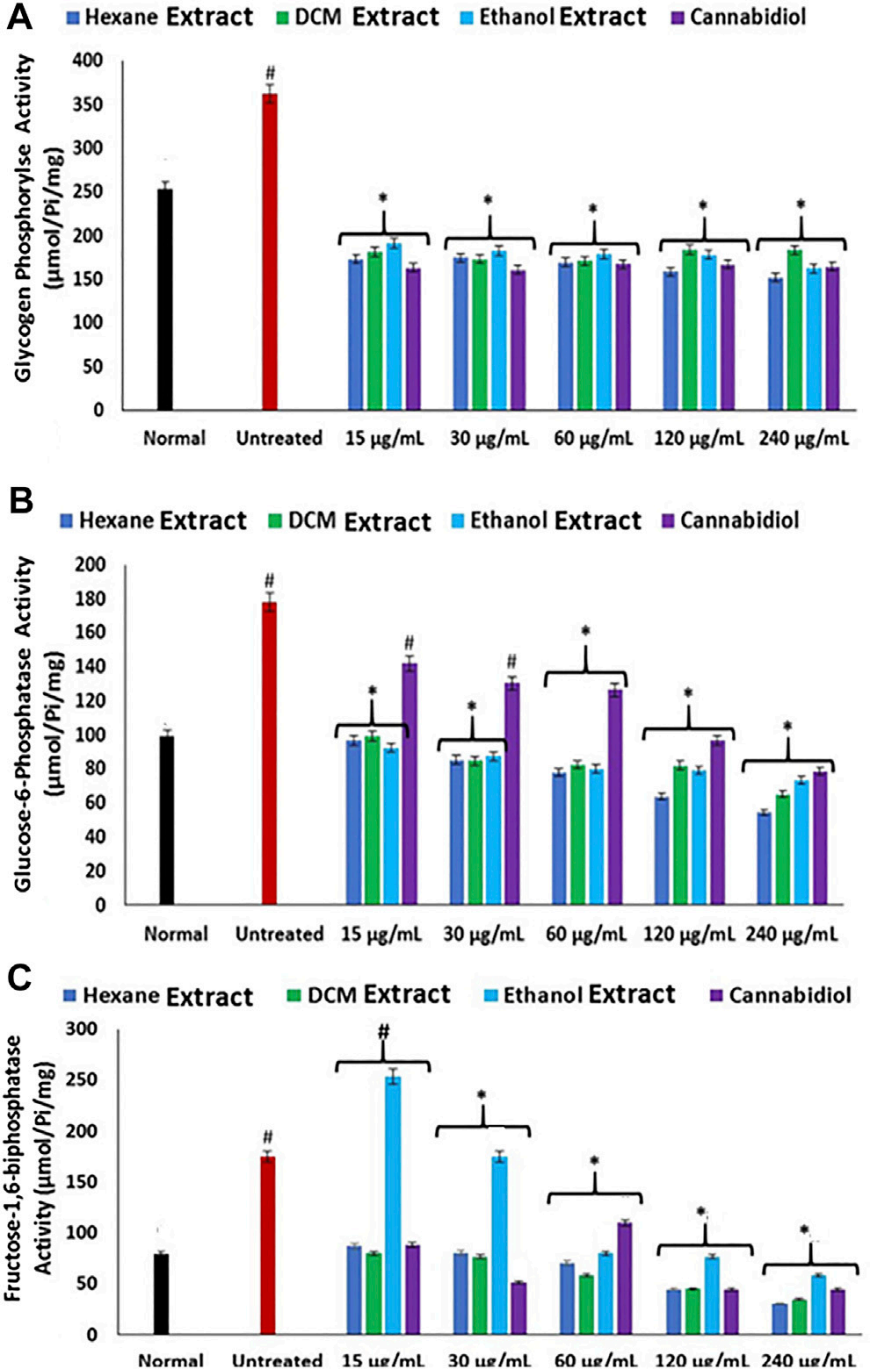
Effect of *Cannabis sativa* on **(A)** glycogen phosphorylase, **(B)** glucose-6-phosphatase, and **(C)** fructose-1,6-bisphosphatase activities in oxidative hepatic injury. Data = mean ± SD; *n* = 3. *Statistically significant (*p* < 0.05) compared with untreated tissues; #statistically significant (*p* < 0.05) compared with normal tissues. Normal: liver tissues not treated with FeSO_4_ and/or *C. sativa*. Untreated: liver tissues treated with FeSO_4_ only.

FeSO_4_-induced oxidative injury significantly elevated the activities of glycogen phosphorylase, glucose-6-phosphatase, and fructose-1,6-bisphosphatase as shown in Figures 5A–C. Elevation of these carbohydrate metabolizing enzymes indicates increased gluconeogenesis, which insinuates elevated hepatic concentration of glucose (Erukainure et al., 2019). Increased gluconeogenesis has been implicated in oxidative injury and recognized as one of the main mechanisms of liver diseases (Teimouri et al., 2006; Sunny et al., 2011). Elevated hepatic glucose levels portray a toxic effect, as surplus glucose can be channeled to the polyol pathway, advanced glycation end-product (AGE) formation, the hexosamine pathway, and protein kinase C (PKC) isoforms, where they serve as fuel (Erukainure, Ochuko L et al., 2019; Luo et al., 2016). Excessive glucose can also undergo oxidation to produce the enediol radical in the presence of transition metal, which breaks down to reactive ketoaldehydes and superoxide anion (O_2_^**.**−^) radicals (Maritim et al., 2003). Treatment with *C. sativa* extracts led to significantly (*p* < 0.05) reverse these enzyme activities, thus indicating a glycogenic effect. The glycolytic effect of the extracts insinuates the reduced availability of glucose for oxidation and the aforementioned oxidative pathways.

**FIGURE 6:**
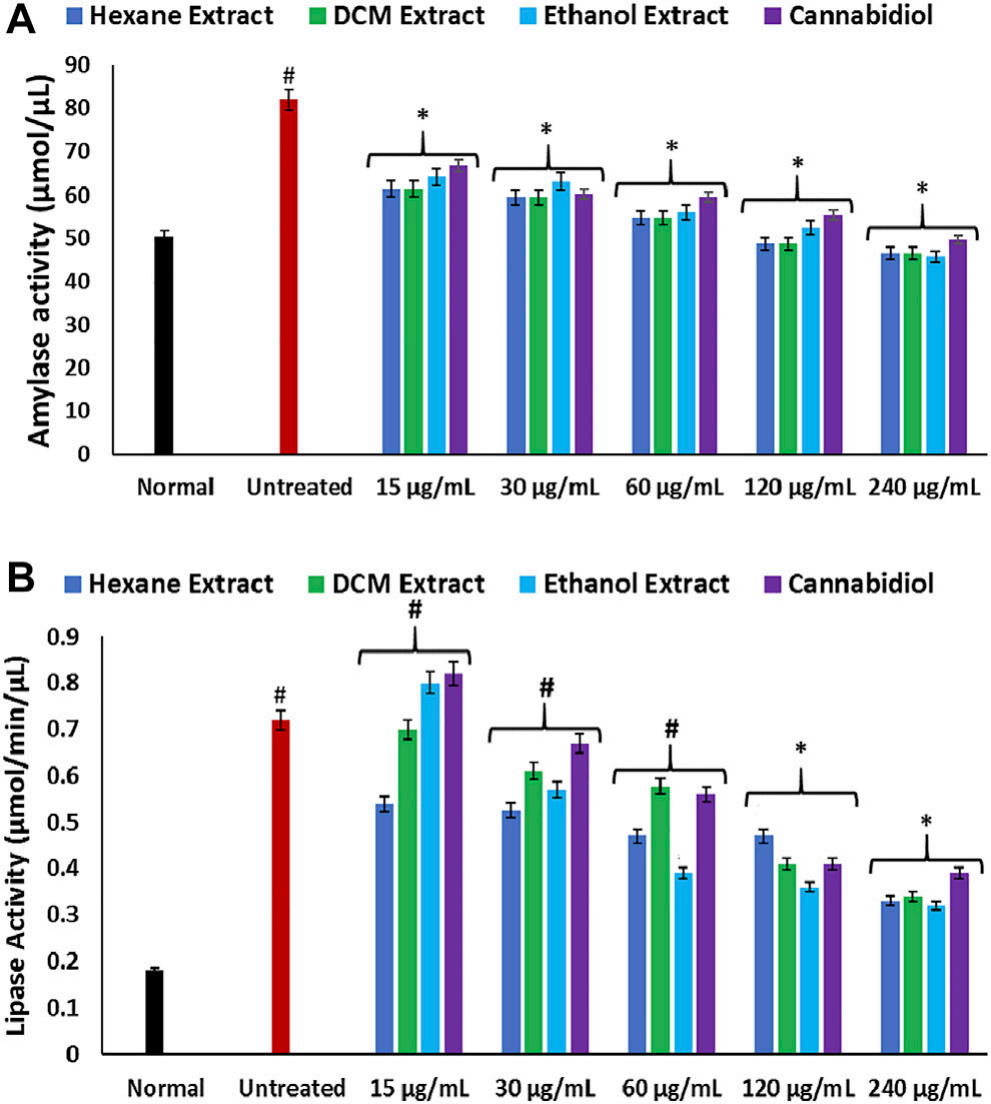
Effect of *Cannabis sativa* on **(A)** amylase and **(B)** lipase activities in oxidative hepatic injury. Data = mean ± SD; *n* = 3. *Statistically significant (*p* < 0.05) compared with untreated tissues; #statistically significant (*p* < 0.05) compared with normal tissues. Normal: liver tissues not treated with FeSO_4_ and/or *C. sativa*. Untreated: liver tissues treated with FeSO_4_ only.

The increased hepatic amylase activity in inducing oxidative injury as depicted in Figure 6A further corroborates an elevated hepatic concentration of glucose in oxidative hepatotoxicity. Amylase catalyzes the disintegration of dietary carbohydrates to glucose and has been implicated in hyperglycemia (Oyebode et al., 2019; Sanni et al., 2019). Induction of oxidative injury also led to elevated activity of hepatic lipase (Figure 6B). Elevated hepatic lipase activity has been implicated in hepatotoxicity as it regulates TG levels, leading to a buildup of lipids in the liver (Bersot et al., 1999; Mahley et al., 2000). Its elevation on induction of oxidative injury therefore insinuates a dysregulated TG metabolism, thus contributing to liver steatosis, which is characterized by increased hepatic lipid accumulation (Kawano and Cohen, 2013). Treatment with *C. sativa* significantly inhibited hepatic amylase and lipase activities, which indicates the ability of the extracts to modulate glucose and lipid accumulations in oxidative hepatotoxicity.

**FIGURE 7:**
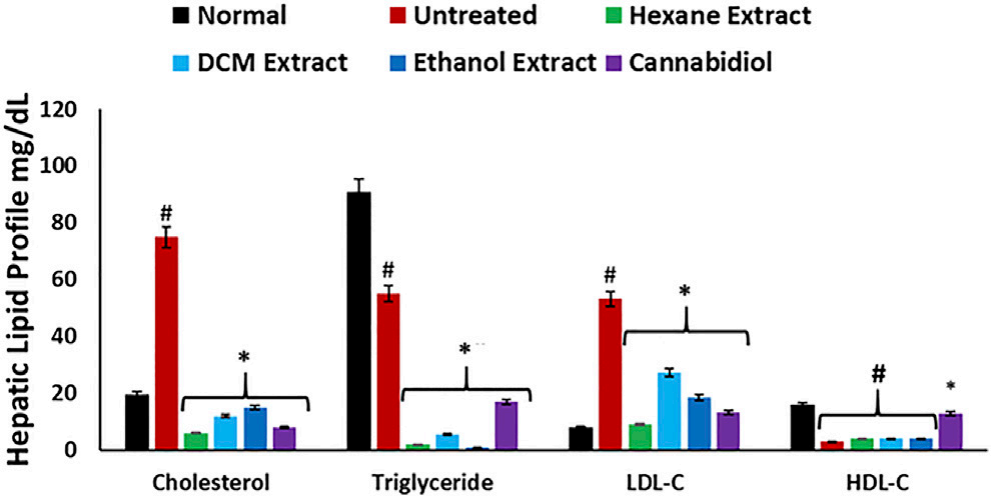
Effect of *Cannabis sativa* on lipid profile in oxidative hepatic injury. Data = mean ± SD; *n* = 3. *Statistically significant (*p* < 0.05) compared with untreated tissues; #statistically significant (*p* < 0.05) compared with normal tissues. Normal: liver tissues not treated with FeSO_4_ and/or *C. sativa*. Untreated: liver tissues treated with FeSO_4_ only.

As shown in Figure 7, induction of oxidative injury instigated significant (*p* < 0.05) elevation of hepatic levels of cholesterol, low-density lipoprotein–cholesterol (LDL-C), with concomitant depleted levels of TG and HDL-C, thereby depicting a dysregulated lipid profile, which is a hallmark of most liver diseases such as hepatitis and cirrhosis (Cheung and Sanyal, 2008; Min et al., 2012). These levels correlate with the increased hepatic lipase activity on induction of oxidative injury (Figure 6B) and insinuate an accumulation of lipids. Treatment with *C. sativa* significantly (*p* < 0.05) reduced hepatic levels of cholesterol and LDL-C but had no effect on the TG and HDL-C levels. The ability of the extracts to deplete hepatic levels of cholesterol and LDL-C may further portray a modulatory effect on hepatic lipid metabolism in oxidative-mediated hepatotoxicity.

**FIGURE 8:**
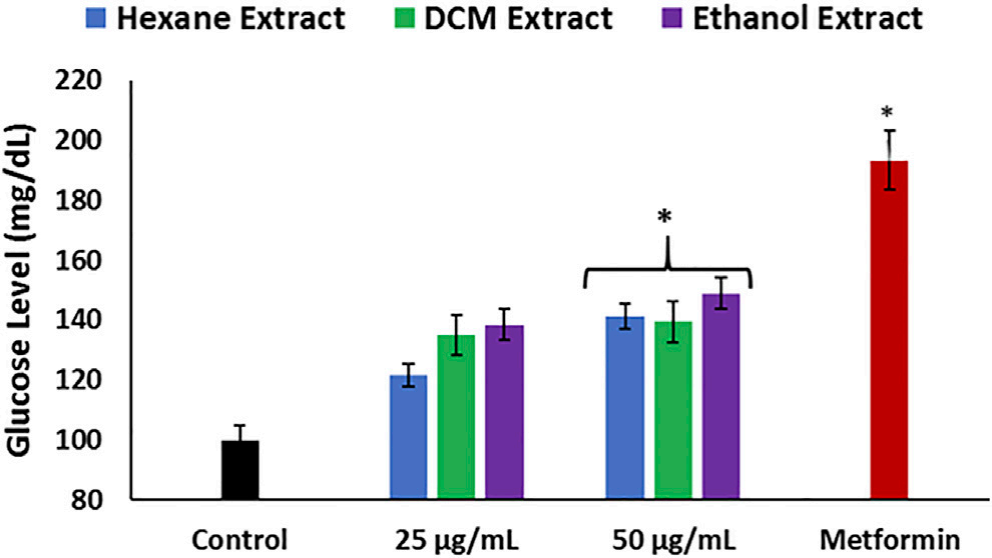
Effect of *Cannabis sativa* on glucose utilization in Chang liver cells. Data = mean ± SD; *n* = 3. *Statistically significant (*p* < 0.05) compared with control.

To further ascertain the effect of *C. sativa* extracts on hepatic glucose metabolism, their ability to stimulate glucose utilization in the liver was determined in Chang liver cells. Impaired hepatic glucose utilization characterized by hepatic glucose intolerance leading to accumulation of glucose has been implicated in the pathogenesis of hepatotoxicity (Riggio et al., 1982; Imano et al., 1999; Merli et al., 1999). All extracts stimulated liver glucose utilization at the highest concentration and were compared favorably with metformin as shown in Figure 8, thus further insinuating the ability of the extracts to improve glucose metabolism, which corroborates decreased gluconeogenic activity (Figures 5A–C).

HPLC analysis of the extract revealed the presence of phenolics in all extracts, with the ethanol extract having the highest number of identified compounds as shown in Table 1. The HPLC fingerprints are presented in Supplementary Figures S1A–C. The presence of CBD was also detected in all the extracts, while Δ-9-THC was identified in the hexane and DCM extracts only. The antioxidative and hepatoprotective properties of phenolics are well established (Madrigal-Santillán et al., 2014; Erukainure et al., 2018). CBD and Δ-9-THC have been recognized as the main phytoconstituent of *C. sativa* and have been reported for their hepatoprotective properties (Dibba et al., 2018; Ismail et al., 2018). Therefore, the studied activities of *C. sativa* extracts may be associated with the synergistic effect of the identified compounds.

**TABLE 1:** HPLC identified compounds in *Cannabis sativa* leaf extracts.

Compounds (retention time)	Parameters	Hexane	DCM	Ethanol
Rutin (1.44 min)	RT (min)	–	–	1.46
	DFS (min)			−0.02
Quercetin (1.78 min)	RT (min)	–	–	1.78
	DFS (min)			0.00
Cinnamic acid (1.93 min)	RT (min)	–	–	1.92
	DFS (min)			0.01
Vanillic acid (1.59 min)	RT (min)	1.57	–	–
	DFS (min)	0.02		
Vanillin (1.84 min)	RT (min)	1.83	1.89	–
	DFS (min)	0.01	−0.05	
Cannabidiol (7.71 min)	RT (min)	7.95	7.91	7.88
	DFS (min)	−0.24	−0.20	−0.17
Delta-9-tetrahydrocannabinol (14.44 min)	RT (min)	14.31	14.33	–
	DFS (min)	0.13	0.11	

Note. RT, retention time; DFS, difference in retention time; DCM, dichloromethane.

**FIGURE 9:**
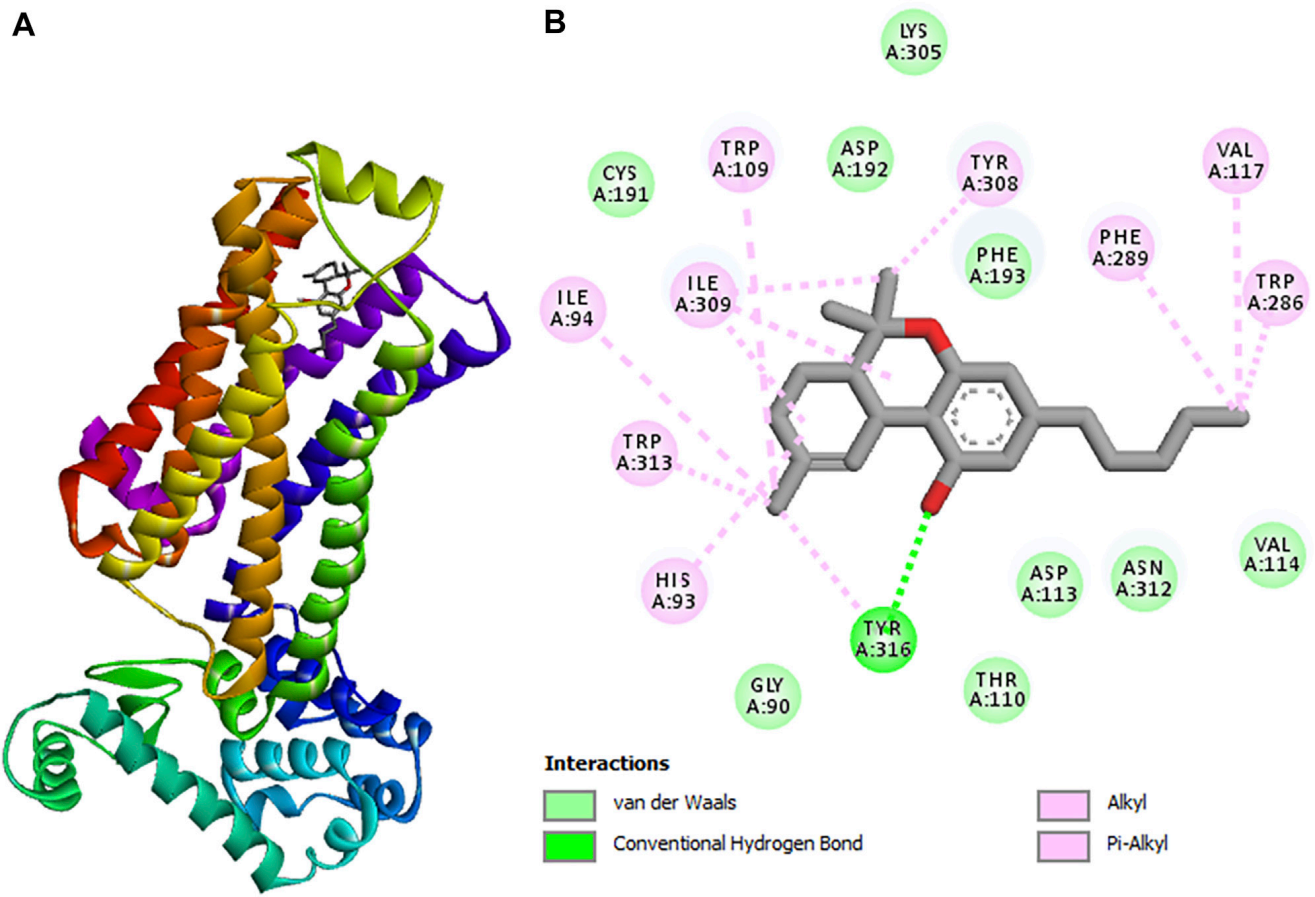
3D structure of **(A)** tetrahydrocannabinol (highest binding affinity) in complex with cannabinoid receptor. **(B)** 2D representations displaying the interactions with amino acid residues.

To understand the role of *C. sativa* extracts in modulating hepatotoxicity, CBD and Δ-9-THC were molecularly docked with the human *β*2 adrenergic receptor. Δ-9-THC showed the best binding potential as revealed by its binding energy of −9.0 kcal/mol as shown in Table 2. It binds to the TYR 316 residue of the human *β*2 adrenergic receptor as depicted in Table 2 and Figure 9, thus insinuating a potent molecular interaction with the *β*2 adrenergic receptor. CBD also showed a potent interaction with a binding energy of −8.7 kcal/mol and bound to TYR 308 and THR 110 residues of the human *β*2 adrenergic receptor (Table 2; Supplementary Figure S2). The *β*2 adrenergic receptor has been implicated in hepatotoxicity, as its activation has been linked to exacerbated hepatic TG, lipogenesis, and dysregulated fatty acid metabolism (Shi et al., 2021). The molecular interactions between the phytocannabinoids and the receptor may therefore indicate the potentials of CBD and Δ-9-THC to inactivate the *β*2 adrenergic receptor, thus modulating the adrenergic system. This may explain the ability of *C. sativa* extracts to inhibit hepatic lipase activity (Figure 6B) and suppress hepatic TG level (Figure 7) in oxidative hepatic injury.

**TABLE 2:** Binding energies and core amino acid residue between residues and ligand from molecular docking.

Compounds	Binding energies (kcal/mol)	Amino acid residues
Tetrahydrocannabinol	−9.0	TYR 316
Cannabidiol	−8.7	TYR 308, THR 110

These results indicate that the induction of oxidative hepatic injury led to oxidative stress (Figure 1), which has been implicated in proinflammation when O_2_^**.**−^ arising from suppressed SOD activity (Figure 1B) reacts with high NO level (Figure 2) to form peroxynitrite radicals. Oxidative stress has also been reported to exacerbate acetylcholinesterase activity (Figure 3), with concomitant dysregulation of lipid metabolism as depicted by the increased lipase activity (Figure 6B) and exacerbated hepatic levels of cholesterol, TG, and LDL-C (Figure 7). Hysterical gluconeogenesis as depicted by the increased activities of glycogen phosphorylase, glucose-6-phosphatase, fructose-1,6-bisphosphatase, and amylase activity (Figures 5A, 6A) results in high hepatic levels of glucose and depleted levels of ATPs and adenosines (Figures 4A,B) in response to low hepatic glucose utilization (Figure 8). Excessive glucose levels arising from hysterical gluconeogenesis may contribute to oxidative stress (Figure 1) *via* oxidation to the enediol radical. Thus, a proposed hepatoprotective mechanism of *C. sativa* against oxidative hepatic injury involves its ability to mitigate oxidative stress (Figure 1), proinflammation (Figure 2), lipid dysmetabolism (Figures 6B, 7), glucose dysmetabolism (Figures 5, 6A), and cholinergic and purinergic dysfunction (Figures 3, 4) while concomitantly stimulating hepatic glucose uptake and utilization (Figure 8).

## Conclusion

The data obtained in this study indicate the ability of *C. sativa* to protect against oxidative-mediated hepatic injury by stalling oxidative stress, gluconeogenesis, and hepatic lipid accumulation while modulating cholinergic and purinergic activities. These activities may be associated with the synergistic effect of the identified phenolics, CBD, and Δ-9-THC and possible interactions with the adrenergic system. However, the present study is the first of several studies we have outlined to investigate the therapeutic effect of *C. sativa* on oxidative-mediated hepatic injury. Further *in vivo* studies to decipher the molecular mechanism behind these activities are currently in place in our lab.

## Data Availability

The original contributions presented in the study are included in the article/supplementary material. Further inquiries can be directed to the corresponding author.

## Abbreviations

ATPase, adenylpyrophosphatase; CBD, cannabidiol; DCM, dichloromethane; ENTPDase, ecto-nucleoside triphosphate diphosphohydrolase; FeSO_4_, iron II sulfate; GSH, reduced glutathione; HDL-C, high-density lipoprotein-cholesterol; HPLC, high-performance liquid chromatography; LDL-C, low-density lipoprotein-cholesterol; MDA, malondialdehyde; NO, nitric oxide; SOD, superoxide dismutase; and Δ-9-THC, Δ-9-tetrahydrocannabinol.
