# Supplementary material for: Cannabis sativa L. (var. indica) Exhibits Hepatoprotective Effects by Modulating Hepatic Lipid Profile and Mitigating Gluconeogenesis and Cholinergic Dysfunction in Oxidative Hepatic Injury
Source: Front Pharmacol. 2021 Dec 21;12:705402. doi: 10.3389/fphar.2021.705402 (PMC8724532; doi:10.3389/fphar.2021.705402)
Supplement: Supplementary file 1 [file DataSheet1.docx]

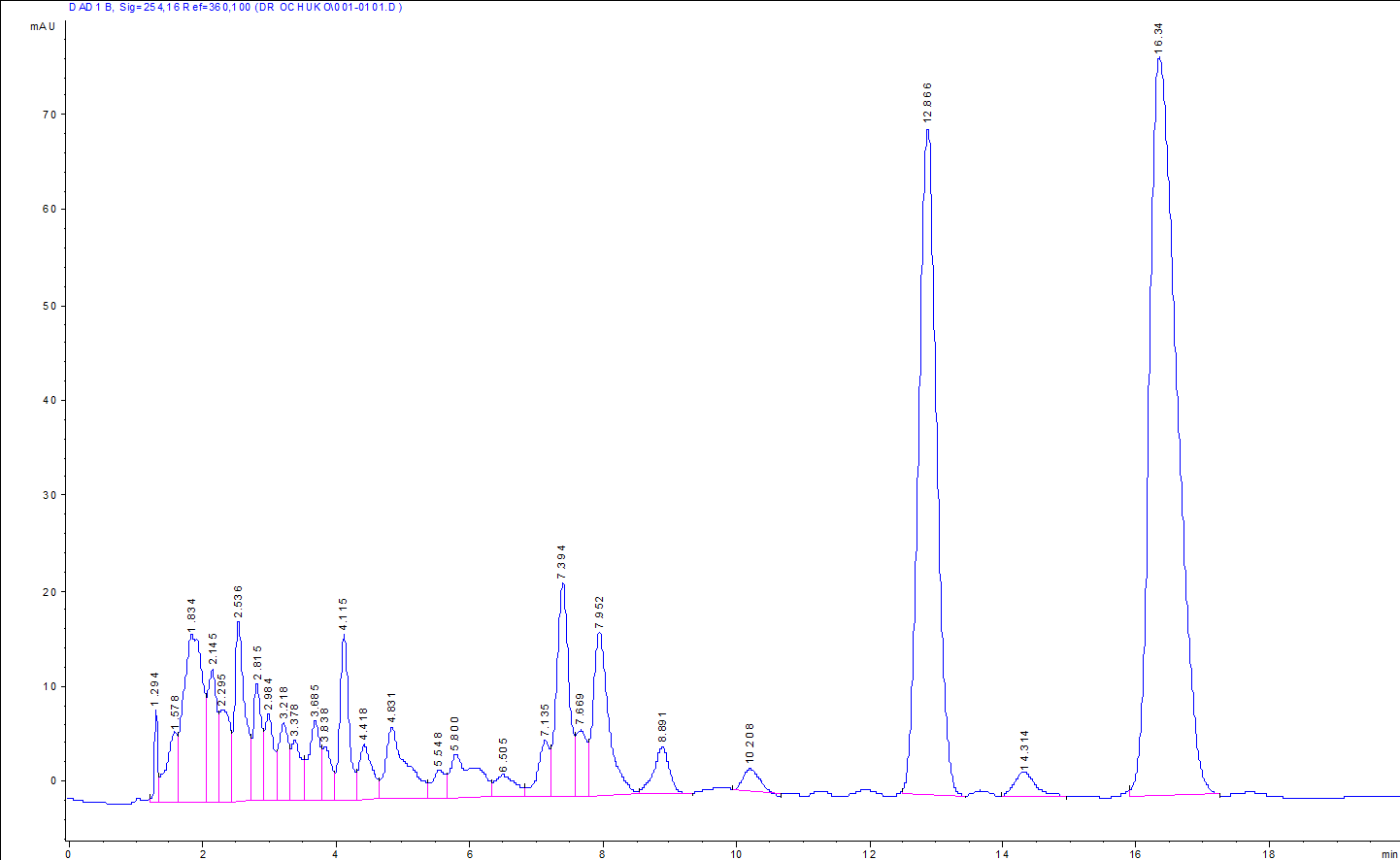


**Fig. S1A**: HPLC fingerprint of hexane extract of *C. sativa*


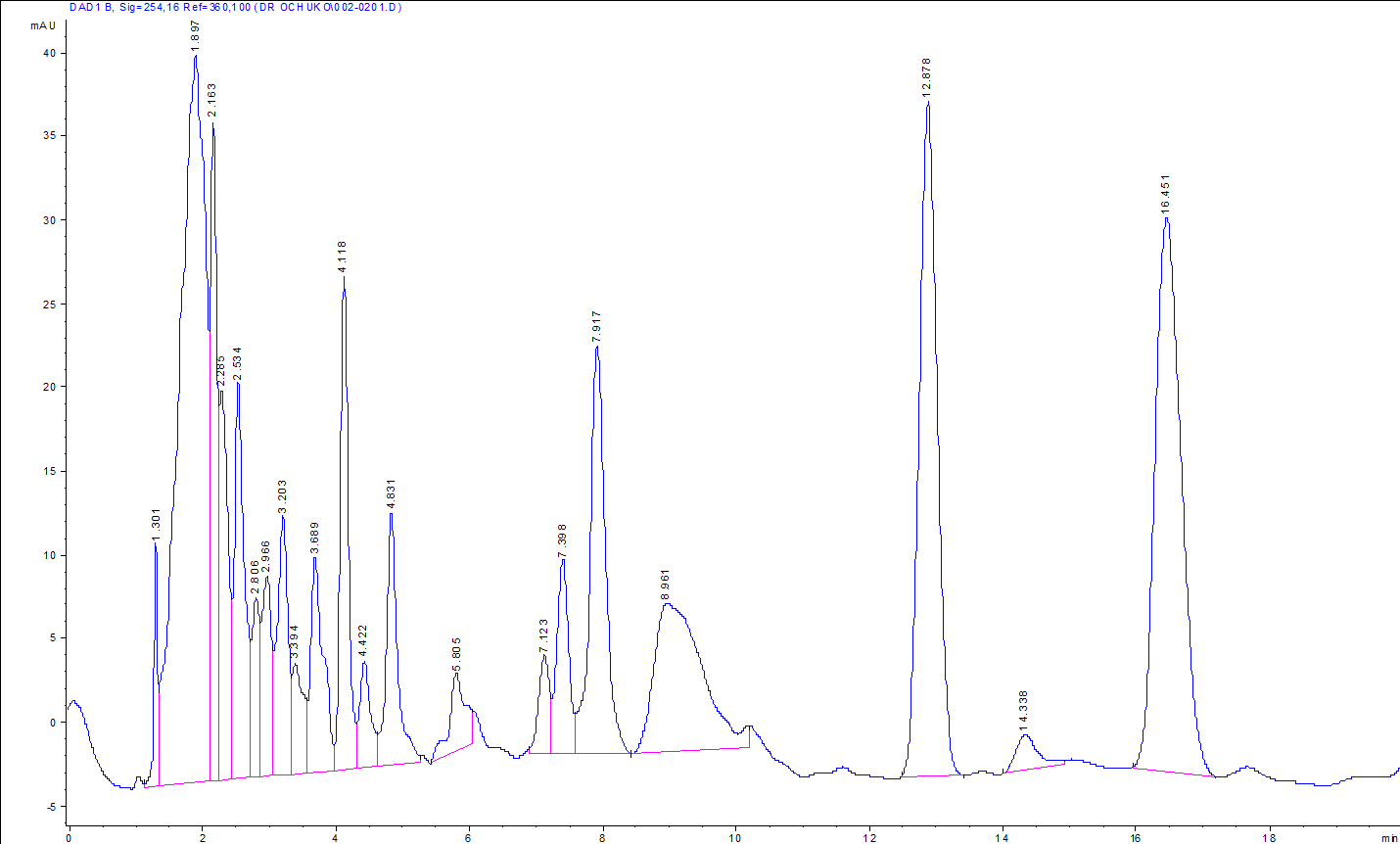


**Fig. S1B**: HPLC fingerprint of DCM extract of *C. sativa*


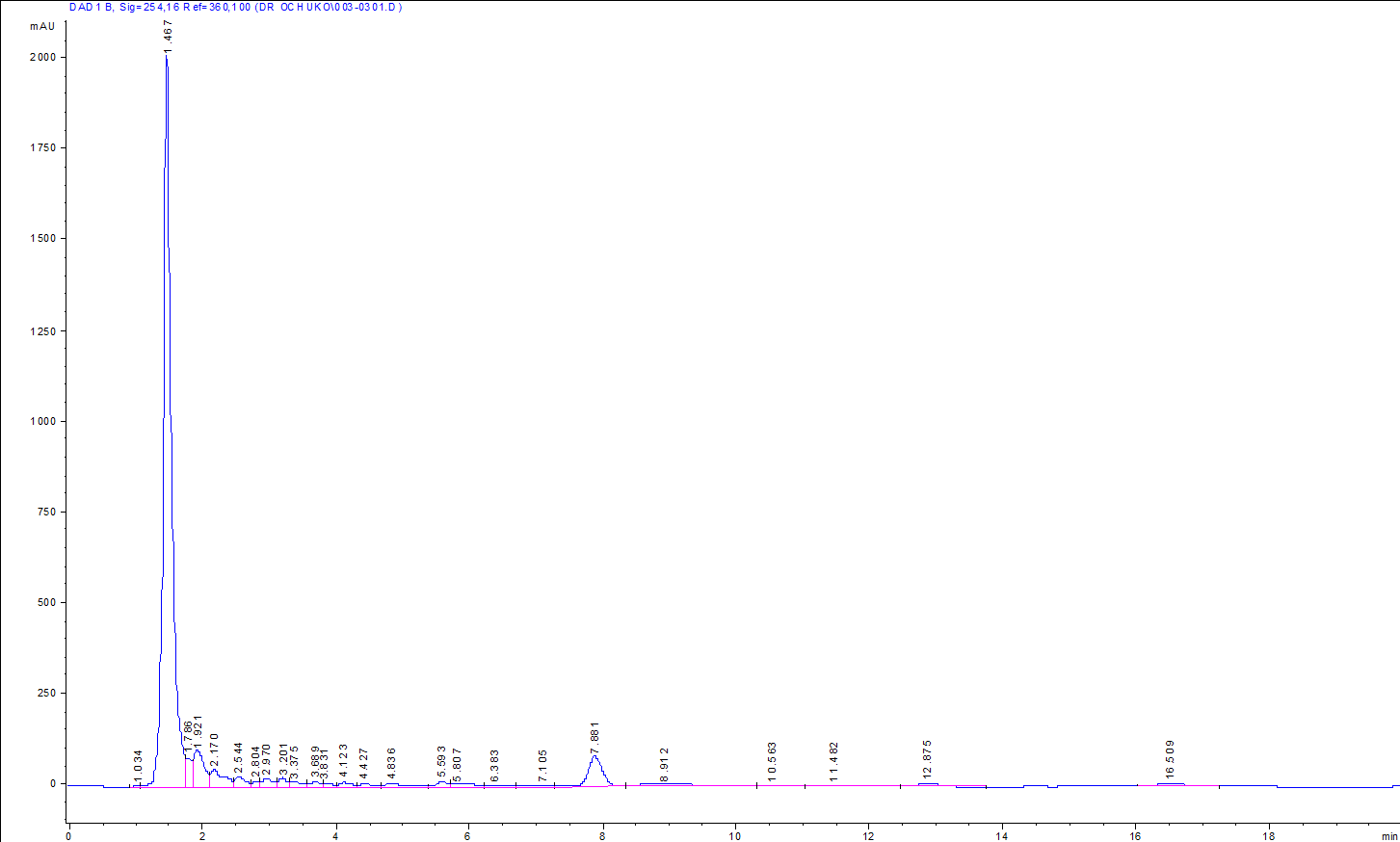


**Fig. S1C**: HPLC fingerprint of ethanol extract of C. sativa


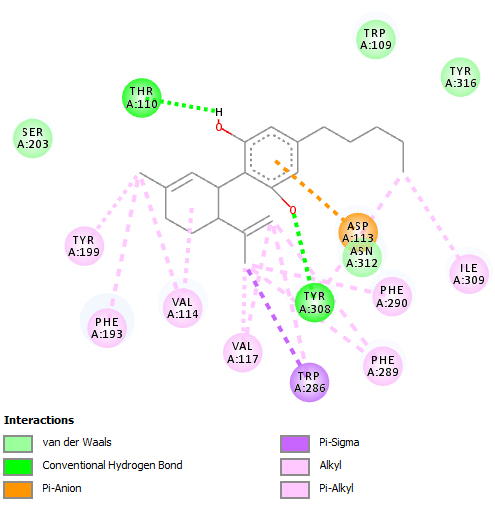


**Fig. S2:** 3D structure of (A) cannabidiol in complex with cannabinoid receptor. (B) 2-D representations displaying the interactions with amino acid residues.
